# Synergistic stimulation of osteoblast differentiation of rat mesenchymal stem cells by leptin and 25(OH)D_3_ is mediated by inhibition of chaperone-mediated autophagy

**DOI:** 10.1186/s13287-021-02623-z

**Published:** 2021-10-30

**Authors:** Qiting He, Ruixi Qin, Julie Glowacki, Shuanhu Zhou, Jie Shi, Shaoyi Wang, Yuan Gao, Lei Cheng

**Affiliations:** 1 Department of Orthopedic Surgery, Qilu Hospital, Cheeloo College of Medicine, Shandong University, 107 Wenhuaxi Road, Jinan, 250012 Shandong People’s Republic of China; 2 Department of Pathology, Qilu Hospital, Cheeloo College of Medicine, Shandong University, Jinan, Shandong People’s Republic of China; 3 Department of Orthopedic Surgery, Brigham and Women’s Hospital, Harvard Medical School, Boston, MA USA; 4 Department of Oral and Maxillofacial Surgery, Harvard School of Dental Medicine, Boston, MA USA

**Keywords:** Bone marrow mesenchymal stem cells, 25(OH)D_3_, Megalin, Osteoblast differentiation, Chaperone-mediated autophagy

## Abstract

**Background:**

Vitamin D is important for the mineralization of bones by stimulating osteoblast differentiation of bone marrow mesenchymal stem cells (BMMSCs). BMMSCs are a target of vitamin D action, and the metabolism of 25(OH)D_3_ to biologically active 1α,25(OH)_2_D_3_ in BMMSCs promotes osteoblastogenesis in an autocrine/paracrine manner. Our previous study with human BMMSCs showed that megalin is required for the 25(OH)D_3_-DBP complex to enter cells and for 25(OH)D_3_ to stimulate osteoblast differentiation in BMMSCs. Furthermore, we reported that leptin up-regulates megalin in those cells. Leptin is a known inhibitor of PI3K/AKT-dependent chaperone-mediated autophagy (CMA). In this study, we tested the hypothesis that leptin acts synergistically with 25(OH)D_3_ to promote osteoblastogenesis in rat BMMSCs by a mechanism that entails inhibition of PI3K/AKT-dependent CMA.

**Methods:**

BMMSCs were isolated from rat bone marrow (4-week-old male SD rats); qRT-PCR and western immunoblots or immunofluorescence were used to evaluate the expression of megalin, ALP, COL1A1, RUNX2, OSX, OSP, and CMA in rBMMSCs. The osteoblast differentiation was evaluated by ALP activity, ALP staining, and calcium deposition. The viability of rBMMSCs was assessed with the CCK-8 kit. Biosynthesis of 1α,25(OH)_2_D_3_ was measured by a Rat 1α,25(OH)_2_D_3_ ELISA Kit.

**Results:**

The combination of leptin and 25(OH)D_3_ treatment significantly enhanced osteoblast differentiation as shown by ALP activity, ALP staining, and calcium deposition, the expression of osteogenic genes ALP, COL1A1, RUNX2, OSX, and OSP by qRT-PCR and western immunoblots in rBMMSCs. Leptin enhanced the expression of megalin and synthesis of 1α,25(OH)_2_D_3_ in rBMMSCs. Our data showed that leptin inhibited CMA activity of rBMMSCs by activating PI3K/AKT signal pathway; the ability of leptin to enhance 25(OH)D_3_ promoted osteoblast differentiation of rBMMSCs was weakened by the PI3K/AKT signal pathway inhibitor.

**Conclusions:**

Our data reveal the mechanism by which leptin and 25(OH)D_3_ promote osteoblast differentiation in rBMMSCs. Leptin promoted the expression of megalin by inhibiting CMA, increased the utilization of 25(OH)D_3_ by rBMMSCs, and enhanced the ability of 25(OH)D_3_ to induce osteoblast differentiation of rBMMSCs. PI3K/AKT is at least partially involved in the regulation of CMA. These data indicate the importance of megalin in BMMSCs for vitamin D’s role in skeletal health.

**Supplementary Information:**

The online version contains supplementary material available at 10.1186/s13287-021-02623-z.

## Introduction

The maintenance of bone mass in the human body results from the dynamic balance of bone resorption and bone formation, osteoclasts absorb calcified bone matrix, and osteoblasts synthesize new bone matrix. When the rate of bone resorption is faster than bone formation, the bone mass decreases gradually, which eventually leads to osteoporosis and increases the risk of fracture, which usually occurs in older people and postmenopausal women as well as in many secondary osteoporosis cases, e.g., cortisone, malnutrition, renal failure, immobilization, side effect of drugs [1–3]. Bone marrow mesenchymal stem cells (BMMSCs) have multilineage differentiation potential to osteoblasts, adipocytes, and chondrocytes in suitable culture environments, proliferate in vitro [4, 5]. MSCs derived from bone marrow and adipose tissue have been studied for the treatment of various diseases, and the paracrine and distant signals of MSCs influence and maintain normal physiological functions of the organs [6]. BMMSCs are widely used in regenerative medicine these years [6]. BMMSCs are the precursors of osteoblasts, but the osteoblast differentiation of BMMSCs involves a series of complex mechanisms, which still need to be studied [7].

Vitamin D is essential for the overall mineralization of bones and the speed of bone conversion [8]. Vitamin D deficiency reduces bone mineral density and increases osteoporosis risk and even generates a propensity to fracture. Bone is one of the important target organs of 1α,25(OH)_2_D_3_, the active metabolite of vitamin D, in which 1α,25(OH)_2_D_3_ induces BMMSCs to differentiate into osteoblasts [9]. 25-Hydroxycholecalciferol (25(OH)D_3_) needs to be hydroxylated to 1α,25(OH)_2_D_3_ by 25(OH)D_3_-1α-hydroxylase before it has a physiological role [10]. 25(OH)D_3_ is more stable than 1α, 25(OH)_2_D_3_ in blood; the half-life of 25(OH)D_3_ is about 3 weeks, and 1α, 25(OH)_2_D_3_ is only about 4 h. Vitamin D mainly in the form of 25(OH)D_3_ exists in the blood, and 25(OH)D_3_ has a high affinity with vitamin D binding protein (DBP) [11–13]. It is reported that BMMSCs contain 25(OH)D_3_-1α-hydroxylase, which hydroxylates 25(OH)D_3_ into the more active form, 1α, 25(OH)_2_D_3_ [33]. The level of 1α-hydroxylase of 25(OH)D_3_ is related to osteogenesis [14, 15].

Our previous studies showed that megalin (lipoprotein-related protein 2; LRP2; gp330) is one of the key receptors for 25(OH)D_3_ into BMMSCs [16]. In vitro, 25(OH)D_3_ entered cells as the 25(OH)D_3_-DBP complex via megalin receptors on the cell membrane and hydroxylated to 1α, 25(OH)_2_D_3_ under the action of 25(OH)D_3_-1α-hydroxylase. We also found that there were significant differences in constitutive expression of megalin in samples from different human subjects [16]. The samples with low expression of megalin were less sensitive to 25(OH)D_3_, which arises the hypothesis in this study that up-regulation of megalin improves to enhance 25(OH)D_3_ to stimulate the osteoblast differentiation of BMMSCs. Leptin directly affects osteoblasts and indirectly affects bone mineral density through the change of body weight, in which leptin levels are higher in obese people, and obesity is positively correlated with bone mineral density [17]. It was found that leptin up-regulates the expression of megalin receptor [16]. The relationship between leptin and 25(OH)D_3_ on osteoblast differentiation remains unclear; in this study, we investigated whether leptin enhances the sensitiveness of BMMSCs to 25(OH)D_3_ by promoting the expression of megalin.

Chaperone-mediated autophagy (CMA) is selective autophagy, which can degrade soluble cellular proteins through the lysosome; the proteins were degraded in the lysosomal cavity through lysosomal-associated membrane protein 2A (LAMP2A), which is the key rate-limiting receptor of CMA and can affect the activity of CMA; various organelle fragments, misfolded proteins, and pathogens are degraded by CMA pathway, and CMA maintains the stable expression of some large proteins such as cell membrane receptors [18]. It is reported that geldanamycin induces CMA to promote the expression of LAMP2A and reduce the expression of ryanodine receptor type-2 in cardiomyocytes [19]. In this study, we investigated how CMA affects the megalin expression and osteoblast differentiation in rBMMSCs.

This study revealed the mechanism by which leptin and 25(OH)D_3_ promote osteoblast differentiation in rBMMSCs via the up-regulation of megalin by inhibiting CMA, increased the utilization of 25(OH)D_3_ by rBMMSCs, and enhanced the ability of 25(OH)D_3_ to induce osteoblast differentiation of rBMMSCs. We demonstrated that PI3K/AKT is at least partially involved in the regulation of CMA. Our data indicate that megalin in BMMSCs is vital for vitamin D’s role in skeletal health.

## Materials and methods

### Ethics statement

All procedures were approved by the Shandong University Committee on the Use and Care of Animals and conducted per the Guidelines for the Care and Use of Laboratory Animals. Sprague Dawley (SD) rats were purchased from SPF (Beijing) Biotechnology Co. Ltd. (Beijing, China).

### Reagents

Recombinant rat leptin (CYT-227) was purchased from Prospec-Tany TechnoGene Ltd (Israel). 25(OH)D_3_ was purchased from Aladdin (Shanghai, China). PI3K/AKT signaling pathway inhibitor LY294002 was purchased from Sigma-Aldrich (Shanghai, China). Antibodies against phosphorylated-p38 (p-p38, #4511), total-p38 (t-p38, #8690), β-actin (#3700), phosphorylated STAT3 (p-STAT3, #9145) and glyceraldehyde-3-phosphate dehydrogenase (GAPDH, #5174), anti-mouse IgG (H + L) (DyLight™ 680 Conjugate or DyLight™ 800 4X PEG Conjugate), and anti-rabbit IgG (H + L) (DyLight™ 680 Conjugate or DyLight™ 800 4X PEG Conjugate) secondary antibody were purchased from Cell Signaling Technology (CST, Shanghai, China). Antibodies against phosphorylated-AKT (p-AKT, sc-293125), total-AKT (t-AKT, sc-81434), total STAT3 (t-STAT3, sc-293151), and megalin (sc-515772) were purchased from Santa Cruz Biotechnology (Shanghai, China). Antibodies against runt-related transcription factor 2 (RUNX2, ab23981), collagen type I (COL1A1, ab6308), and LAMP2A (ab125068) were obtained from Abcam (Abcam, Shanghai, China). Antibodies against HSC70 (AF5187) and leptin receptor (DF7139) were obtained from Affinity Biosciences (Jiangsu, China). Fluorescent second antibody rhodamine (TRITC)-conjugated goat anti-mouse IgG (H + 2) was purchased from ZSGB-BIO (Beijing, China). Alkaline phosphatase (ALP) activity kit, BCIP/NBT alkaline phosphatase (ALP) color development kit, enhanced cell counting Kit-8 (CCK-8), and Alizarin Red Staining (ARS) were purchased from Beyotime (Shanghai, China). Dexamethasone, β-glycerophosphate, and ascorbate-2-phosphate were purchased from Solarbio Science and Technology Co., Ltd. (Beijing, China). Rat 1α,25(OH)_2_D_3_ ELISA Kit was purchased from Bioswamp Life Science Lab (Wuhan, China). All primers were synthesized by BioSune (Shanghai, China).

### Isolation and culture of primary rBMMSCs

Four-week-old (100–150 g) male SD rats were selected as the source of primary rBMMSCs for culture in vitro. The rBMMSCs were isolated from bone marrow with the method as follows. SD rats were euthanized with pentobarbital sodium (35 mg/kg, intraperitoneal injection) followed by cervical dislocation and bathed in 75% ethyl alcohol for 15 min. The muscles were removed from all femurs and tibias, and the bones were soaked and rinsed with aseptic phosphate-buffered saline (PBS; Beijing Dingguo Changsheng Biotechnology, China). Both epiphyses were removed to expose the marrow cavity. The contents of the marrow were flushed into a sterile 100-mm tissue culture dish (NEST Biotechnology Co.LTD, Jiangsu, China) with Dulbecco’s modified Eagle’s medium (DMEM; Gibco) supplemented with 10% fetal bovine serum (Gibco) and 1% penicillin/streptomycin (Gibco). The flushing was repeated 3 times; the process was repeated in the opposite direction. The pooled mixture was centrifuged at 1000 RPM for 5 min, the supernatant was discarded, and the cells were suspended with a complete culture medium and plated into a 100-mm tissue culture dish (NEST Biotechnology Co.LTD). The rBMMSCs were cultured in a saturated humidity incubator with a volume fraction of 5% CO_2_ at 37 °C. Nonadherent cells were removed by medium change after 48 h to 72 h; then, the medium was replaced every 3 days. When the cell density reached 80–90% confluency, the cells were trypsinized and subcultured at the ratio of 1: 3. Passage (P) 3 to 5 rBMMSCs were used in this study.

### RNA extraction and quantitative real-time polymerase chain reaction (qRT-PCR)

The total RNA was extracted from rBMMSCs with TRIzol reagent (SparkJade, Shandong, China) according to the manufacturer’s instructions. Complementary DNA was synthesized with ReverTra Ace Qpcr RT Kit (Toyobo Life Science, Shanghai, China) according to the manufacturer’s instructions. The qRT-PCR was performed on ABI 7900HT Fast Real-Time PCR system (Applied Biosystems, USA) using SYBR Green Realtime PCR Master Mix (Toyobo Life Science) to evaluate the expression levels of megalin, RUNX2, ALP, COL1A1, OSX, OSP, LAMP2A, HSC70, and β-actin (internal control). The 2^−△△Ct^ method was used to calculate gene relative expression levels. The primers for qRT-PCR are shown in Table 1.

**Table 1:** Sequences of primers

Gene	Forward (5′–3′)	Reverse (5′–3′)
Megalin	ACAACTCGGATGAACGGGAC	AGTAGGTGCCGTTGGGAAAG
ALP	AGATGGATGAGGCCATCGGA	CCAAACGTGAAAACGTGGGA
COL1A1	CACTGCAAGAACAGCGTAGC	AAGTTCCGGTGTGACTCGTG
RUNX2	CAGACCAGCAGCACTCCATA	AGACTCATCCATTCTGCCGC
OSX	GGTCCTGGCAACACTCCTAC	AAGAGGTGGGGTGCTGGATA
OSP	GAGACCATGCAGAGAGCGAG	TTGACCTCAGTCCGTAAGCC
LAMP2A	AAGAGCAGGTGGTTTCCGTG	ATGGGCACAAGGAAGTTGTCT
HSC70	CTCCATTACCCGTGCTCGAT	GAACCACCCACCAGGACAAT
LepR	TTGGTCCTCTTCTGGAGC	CCCCTTGTGGAATCTGGAGTG
β-actin	CTCTGTGTGGATTGGTGGCT	CGCAGCTCAGTAACAGTCCG

*ALP* alkaline phosphatase, *COL1A1* collagen type I, *RUNX2* runt-related transcription factor 2, *OSX* osterix, *OSP* osteopontin, *LAMP2A* lysosomal-associated membrane protein 2A, *HSC70* heat-shock cognate 71 kDa protein, *LepR* leptin receptor

### Western immunoblots

Total protein was extracted from rBMMSCs with radioimmunoprecipitation assay lysis buffer (Beyotime) and protease inhibitor cocktail (MedChemExpress, Shanghai, China). Protein concentration was measured with the BCA kit (Boster Biological Technology, Wuhan, China). After denaturation, 40 ug aliquots of protein were separated by sodium dodecyl sulfate-polyacrylamide gel electrophoresis (SDS-PAGE) and transferred to a polyvinylidene difluoride membrane (Merck Millipore, Shanghai, China). Membranes were blocked with 5% nonfat milk at room temperature for 1–2 h. Membranes were sequentially incubated overnight at 4 °C with the following primary antibodies: GAPDH (1:2000; CST), β-actin (1:10,000; CST), RUNX2 (1:1000; Abcam), COL1A1 (1:1000; Abcam), t-AKT (1:400; Santa Cruz Biotechnology), p-AKT (1:1000; Santa Cruz Biotechnology), t-p38 (1:1000; CST), p-p38 (1:1000; CST), p-STAT3 (1:1000; CST), t-STAT3 (1:400; Santa Cruz Biotechnology), LAMP2A (1:2000; Abcam), HSC70 (1:1000; Affinity Biosciences), or leptin receptor (1:1000; Affinity Biosciences). The membranes were washed with TBST three times and incubated with the anti-mouse IgG (H + L) (DyLight™ 680 Conjugate or DyLight™ 800 4X PEG Conjugate) or anti-rabbit IgG (H + L) (DyLight™ 680 Conjugate or DyLight™ 800 4X PEG Conjugate) secondary antibody for 30 min at room temperature. The fluorescent signal on membranes was detected by Odyssey two-color infrared fluorescence imaging system (LI-COR, USA); band gray values were quantified with ImageJ software.

### Cell viability

Effects of leptin and/or25(OH)D_3_ on the viability of rBMMSCs were assessed with the CCK-8 kit (Beyotime). Different concentrations of leptin (0, 50, 100, 300, 500, 800, 1000 ng/ml), 25(OH)D_3_ (0, 50, 100, 300, 500, 750, 1000 nM), or the combination of leptin (300 ng/ml) and 25(OH)D_3_ (100 nM) was added to each well, with 5 replicate wells, at 24 h after seeding in 96-well plates (5000 cells/well). After 24, 48, and 72 h, 10 ul CCK-8 solution was added to each well according to the manufacturer’s instructions. Absorbance at 450 nm was measured with Varioskan flash (Thermo Scientific, USA).

### Osteoblast differentiation of rBMMSCs

The rBMMSCs were plated into 6-well plates (2 × 10^5^/well); after reaching confluence in basal medium, cultures were changed to osteoblast differentiation medium (ODM; basal medium with 10% FBS, 10 nM dexamethasone, 5 mM β-glycerophosphate, and 50 μg/ml ascorbate-2-phosphate); leptin (300 ng/ml) and/or 25(OH)D_3_ (100 nM) were added to the ODM, 24 h after medium changes. The concentration of 25(OH)D_3_ used in the present study was chosen according to the optimal physiological concentrations [20]. The ODM was replaced twice weekly.

### ALP staining and activity assay

ALP staining and activity assays were performed in rBMMSCs in 12-well plates with/without leptin (300 ng/ml) and/or 25(OH)D_3_ (100 nM), 7 days in ODM. For ALP staining, the cells were washed with PBS 3 times and fixed with 4% paraformaldehyde (Solarbio Life Sciences) for 20 min. They were washed 3 times with PBS and stained with BCIP/NBT ALP color development kit (Beyotime). For the measurement of ALP activity, the rBMMSCs were collected and incubated at 37 °C for 30 min according to the manufacturer’s instructions of ALP Activity Assay Kit (Beyotime). The ALP activity was detected at 405 nm with Varioskan flash (Thermo Scientific).

### Assessment of calcium deposition

After the rBMMSCs were cultures with/without leptin (300 ng/ml) and/or 25(OH)D_3_ (100 nM), 21 days in ODM, calcium deposition was quantified with the ARS Kit (Beyotime). In brief, cells were washed 3 times with distilled water, fixed with 4% paraformaldehyde (Solarbio) for 20 min, and washed 3 times with distilled water. Cells were then incubated with 1% ARS solution for 30 min at room temperature and rinsed with distilled water. For measure of the relative value of ARS, the stain was dissolved with 10% cetylpyridinium chloride (Sangon Biotech, Shanghai, China) for 1 h. Aliquots (200 μl) were transferred to 96-well plates and detected at 405 nm with Varioskan flash (Thermo Scientific).

### Immunofluorescence (IF) analysis of cells

The rBMMSCs (5 × 10^4^/well in 24-well plates) were seeded onto alcohol-washed coverslips (diameter: 14 mm, NEST) with 500 ul basal medium; after reaching 60–70% confluence, mediums were changed to ODM with different concentrations of leptin (0, 50, 100, 300 ng/ml), 25(OH)D_3_ (100 nM), and leptin (300 ng/ml) + 25(OH)D_3_ (100 nM) for 24 h. The coverslips were washed with PBS 3 times, each time for 3 min; then, rBMMSCs were fixed with 4% paraformaldehyde for 20 min at room temperature and were washed with PBS 3 times, each time for 3 min. Triton Xmuri 100 (0.2% in PBS, 20 min, room temperature) was used to permeate the cells. They were washed 3 times with PBS and blocked with 5% BSA for 30 min at room temperature. First megalin antibody (1:80; Santa Cruz Biotechnology) was applied to each coverslip and incubated overnight at 4 °C in a humidified chamber. Thereafter, they were washed 3 times and incubated with fluorescent second antibody rhodamine (TRITC)-conjugated goat anti-mouse IgG (H + 2) (1:50; ZSGB-BIO), in the humidified chamber at room temperature for 1 h. Subsequent steps were carried out in darkness. The coverslips were washed with PBS 3 times for 3 min each time. For staining nuclei, the coverslips were incubated with 2-(4-amidinophenyl)-6-indolecarbamidine dihydrochloride (DAPI; Beyotime) for 5 min; images were collected with the Lionheart FX (BioTek, USA). The optical density of more than 6 cells was randomly measured with ImageJ software, and the average optical density was calculated to represent the fluorescence intensity.

### ELISA assay of biosynthesis of 1α,25(OH)_2_D_3_ in rBMMSCs

The rBMMSCs were cultured in 6-well plates in growth medium until confluent; 24 h after the medium was changed to ODM with 25(OH)D_3_ (100 nM), with or without leptin (300 ng/ml), supernatants were collected and stored at − 80 °C for subsequent determination of 1α,25(OH)_2_D_3_. The cells were collected for western immunoblotting. 1α,25(OH)_2_D_3_ levels in the supernatants were measured by a Rat 1α,25(OH)_2_D_3_ ELISA Kit (Bio-Swamp, Wuhan, China) according to the manufactures’ protocol.

### Statistical analysis

All experiments were repeated at least three times. All data are presented as means ± standard deviation, and statistical analyses were performed with SPSS 20. Significant differences between the groups were determined by Student’s *t* test, one-way ANOVA test, and Tukey’s multiple comparisons test, and the Kolmogorov–Smirnov test was used to analyze the normal distribution of data. A value of *P* < 0.05 was considered significant.

## Results

### Identification of rBMMSCs and morphological and microstructure changes during osteoblast differentiation

The spindle shape of P3 rBMMSCs was observed under microscope (see Additional file 1a). We used flow cytometry to detect the purity of P3 rBMMSCs, and the results showed that the positive surface markers CD29, CD90, and CD44 of rBMMSCs were, respectively, 98.31%, 99.37%, 85.66%, and the negative marker CD45 expressed very low (0.16%) (see Additional file 1b). The spindle shape of rBMMSCs changed to irregular shape at day 10 when exposed to leptin and 25(OH)D_3_ treatment during osteoblast differentiation (see Additional file 1c). At the same time, transmission electron microscope showed the microstructure changes of rBMMSCs, the villi of the cell membrane decreased, and the mitochondria swelled (see Additional file 1d).

### Leptin enhanced the expression of megalin in rBMMSCs

**Fig. 1:**
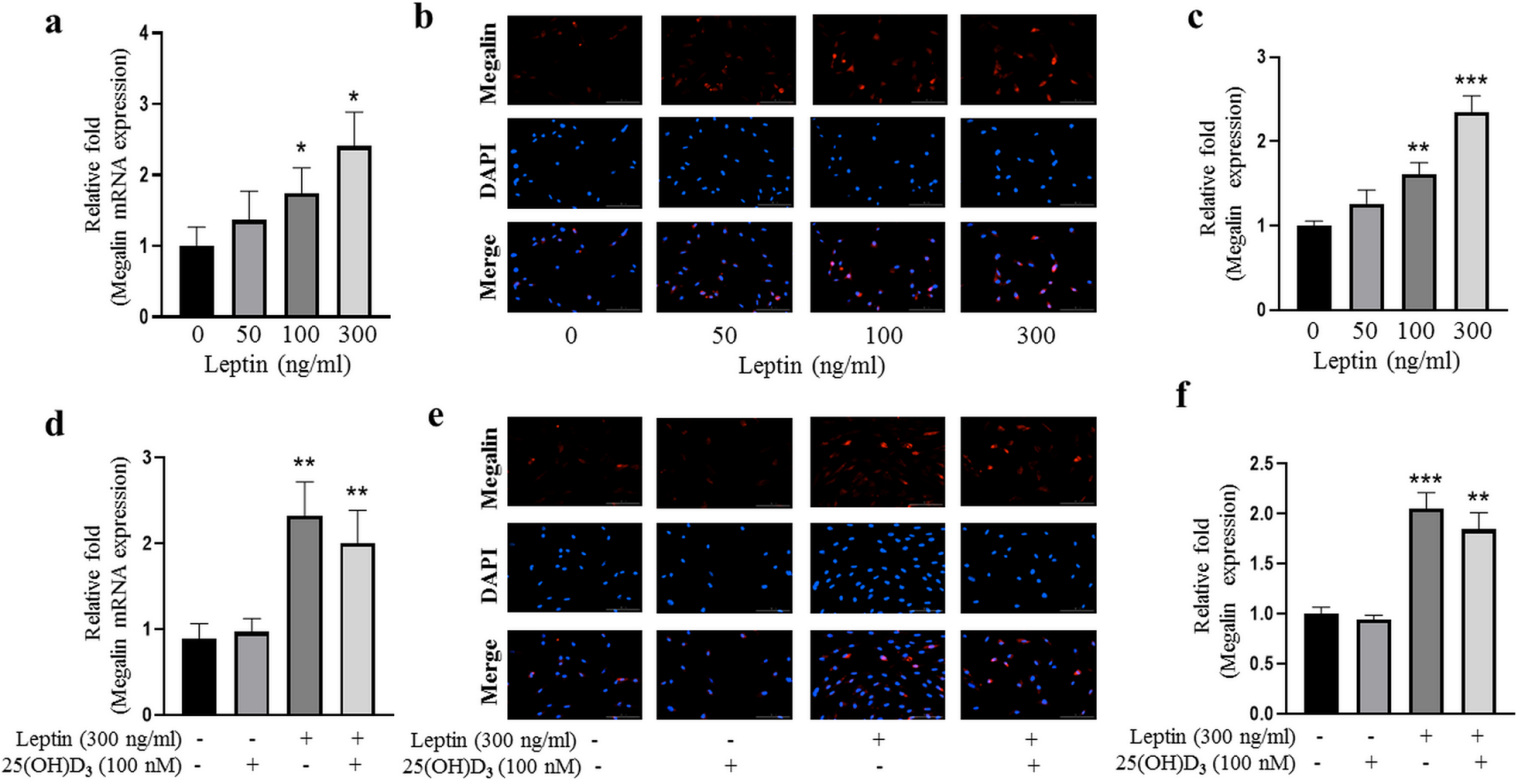
Leptin enhanced the expression of megalin of rBMMSCs. **a** Relative mRNA expression of rBMMSCs megalin in growth medium at 8 h; **b** IF showed leptin enhanced the protein expression of rBMMSCs megalin; **c** relative quantitative analysis of megalin IF staining; **d** 25(OH)D_3_ had no effect on the relative mRNA expression of megalin with or without leptin in osteoblast differentiation medium (ODM) after 8 h; **e** IF showed 25(OH)D_3_ did not affect the protein expression of megalin; **f** relative quantitative analysis of megalin IF staining. (Scale bars = 100 μm. *P < 0.05, **P < 0.01, ***P < 0.001 in comparison with the control group.)

To determine whether leptin and 25(OH)D_3_ promote the expression of megalin in rBMMSCs, qRT-PCR and IF were performed to assess the relative expression levels of megalin mRNA and protein at 8 h and 24 h after treatments, respectively. Our results showed that the expression of megalin in rBMMSCs was dose-dependently up-regulated by leptin (Fig. 1a). At the dose of 300 ng/ml, leptin significantly stimulated megalin expression, and this concentration was used for the follow-up experiments. 25(OH)D_3_ had no significant effect on the expression of megalin with or without leptin, and there was no synergistic or inhibitory effect in combination with leptin (Fig. 1d). IF data confirmed the above qRT-PCR results (Fig. 1b, c, e, f).

### Effects of leptin and/or 25(OH)D_3_ on BMMSCs proliferation

**Fig. 2:**
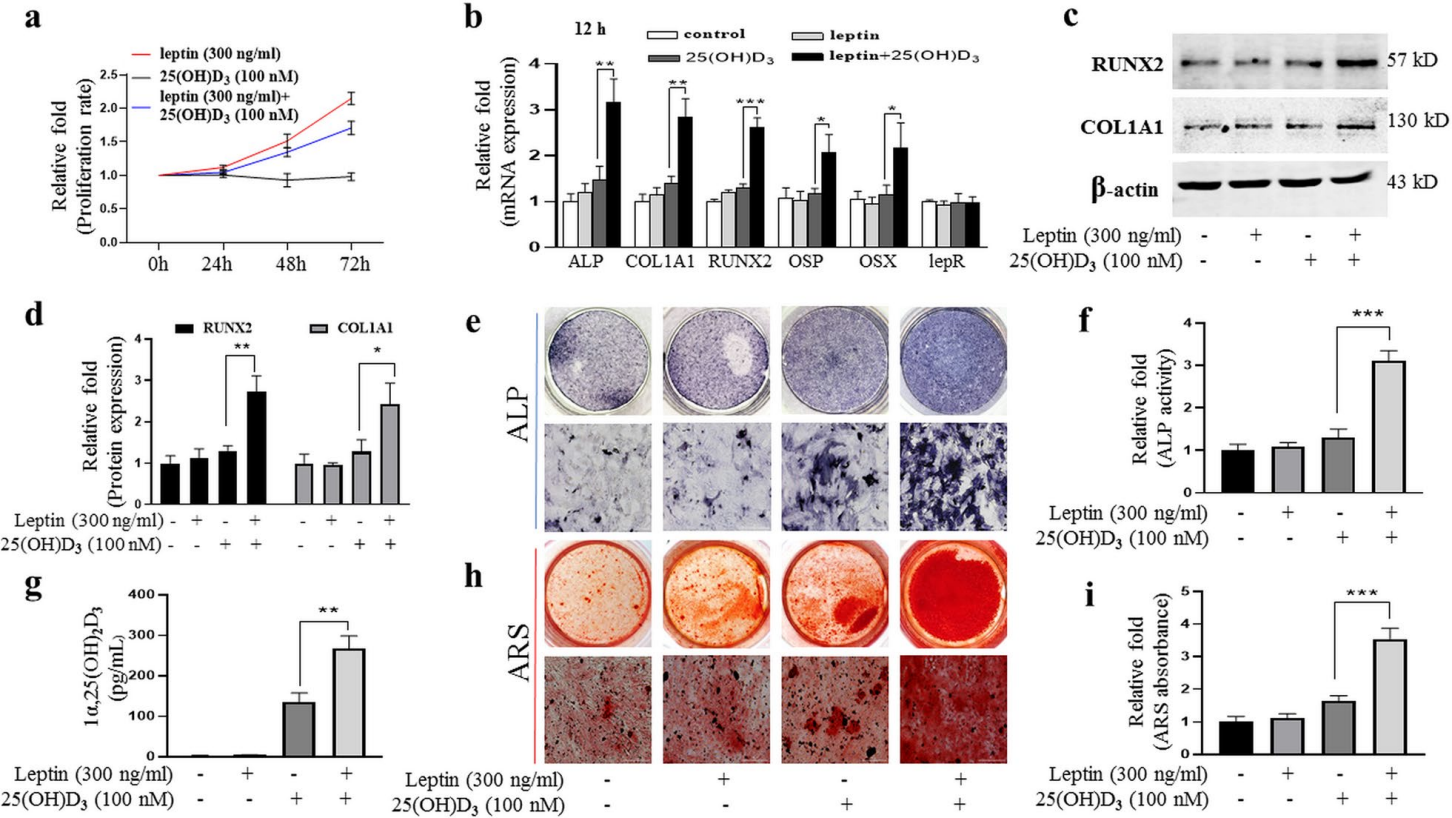
Leptin synergistically with 25(OH)D_3_ to induce osteoblast differentiation of rBMMSCs. **a** The effects of leptin or 25(OH)D_3_ on rBMMSCs proliferation at 24 h, 48 h, and 72 h; **b** relative mRNA expression of osteogenic genes (ALP, COL1A1, RUNX2, OSX, and OSP) at 12 h of osteoblast differentiation; **c** western blot bands of proteins of RUNX2 and COL1A1 at day 3 of rBMMSCs osteoblast differentiation; **d** relative quantitative analysis of proteins of RUNX2 and COL1A1; **e** results of ALP staining of osteoblast differentiation of rBMMSCs at day 7; **f** relative ALP activity fold of osteoblast differentiation of rBMMSCs at day 7; **g** the synthesis of 1α,25(OH)_2_D_3_ by rBMMSCs with ODM at 24 h; **h** results of ARS staining of osteoblast differentiation of rBMMSCs at day 21; **i** relative quantitative analysis of ARS of osteoblast differentiation of rBMMSCs at day 21. (Scale bars = 200 μm. *P < 0.05, **P < 0.01, ***P < 0.001 in comparison with the control group.)

To assess whether leptin and/or 25(OH)D_3_ had cytotoxicity on proliferation/viability of rBMMSCs, different concentrations of leptin and/or 25(OH)D_3_ were used to stimulate rBMMSCs for 3 days, and then, CCK-8 analysis was used to measure cell proliferation/viability. The results showed that leptin at concentrations (50, 800, 1000 ng/ml) has no effect on the proliferation of rBMMSCs at day 3 and, however, promoted the proliferation of rBMMSCs at doses of 100, 300, 500 ng/ml (see Additional file 2a). 25(OH)D_3_ significantly had cytotoxicity on cell proliferation at high doses of 500, 750, 1000 nM alone, but no effect on the proliferation of rBMMSCs at lower doses of 50, 100, 300 nM alone (see Additional file 2b). Moreover, leptin (300 ng/ml) with 25(OH)D_3_ (100 nM) promoted the proliferation of rBMMSCs at 48 h and 72 h (Fig. 2a).

### Leptin synergistically with 25(OH)D_3_ to induce osteoblast differentiation of rBMMSCs

To test whether leptin has a synergistic effect on osteoblast differentiation of rBMMSCs with 25(OH)D_3_, we measured mRNA expression level of osteoblast genes (ALP, COL1A1, RUNX2, OSP, OSX), 12 h, 3, and 5 days after rBMMSCs were transferred to ODM (Fig. 2b, Additional file 3a, b) and also detected the expression level of osteoblast proteins, 3 days after treatments (Fig. 2c, d). The results showed that leptin or 25(OH)D_3_ used alone had no significant effect on osteoblast differentiation of rBMMSCs at the experimental concentrations, but osteoblast differentiation was significantly enhanced when a combination of two agents at 12 h, 3, and 5 days, synergistic effect of leptin and 25(OH)D_3_ was gradually increased with the extension of time. Moreover, leptin or 25(OH)D_3_ had no significant effect on the mRNA and protein expression of leptin receptors (Fig. 2b, Additional file 3a–d). The synthesis of 1α,25(OH)_2_D_3_ was increased significantly by leptin and 25(OH)D_3_ compared with 25(OH)D_3_ group in ODM for 24 h, as assessed by ELISA (Fig. 2g).

### 25(OH)D_3_ and leptin enhanced ALP activity and calcium deposit formation

ALP Activity Assay Kit and ALP staining were used to evaluate the effects of 25(OH)D_3_ with or without leptin on the ALP activity of rBMMSCs. The results showed that there was no significant change in ALP Activity of rBMMSCs at 7 days after 25(OH)D_3_ or leptin was added to ODM alone. The combination of 25(OH)D_3_ and leptin resulted in significantly increased ALP at day 7 (Fig. 2e, f). Similar results were obtained from ARS and relative quantification after 21 days of 25(OH)D_3_ and/or leptin treatments (Fig. 2h, i). These results demonstrated that leptin enhanced synergistically with 25(OH)D_3_ osteoblastic differentiation in rBMMSCs.

### Leptin inhibited CMA of rBMMSCs

**Fig. 3:**
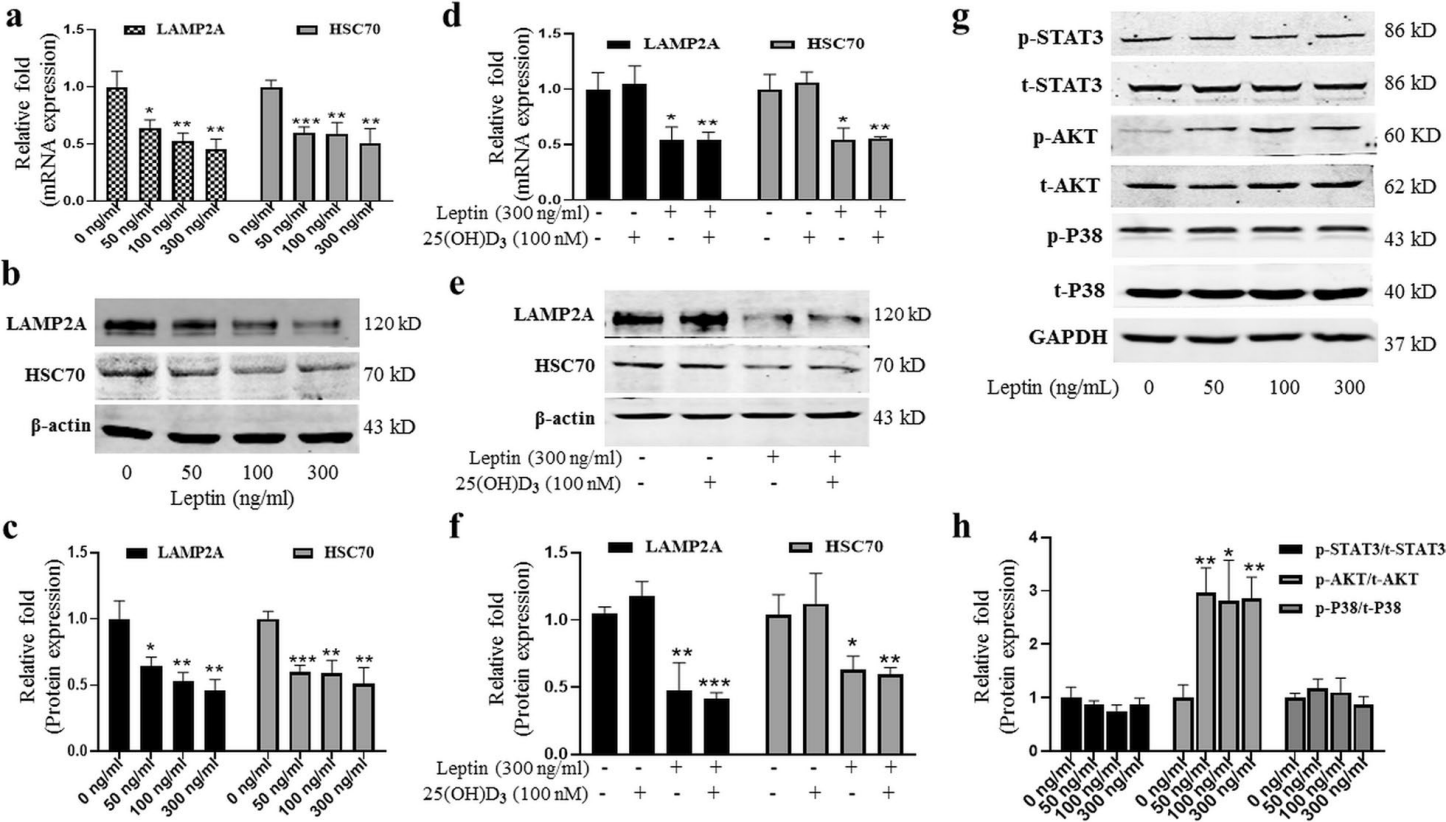
Leptin inhibited CMA of rBMMSCs by activating PI3K/AKT signal pathway. **a** Relative mRNA expression of CMA-related genes (LAMP2A and HSC70) of rBMMSCs in growth medium at 8 h; **b** western blot bands of proteins of LAMP2A and HSC70 of rBMMSCs in growth medium at 24 h; **c** relative quantitative analysis of proteins of LAMP2A and HSC70; **d** 25(OH)D_3_ with or without leptin did not affect the relative mRNA expression of LAMP2A and HSC70 in ODM at 8 h; **e** 25(OH)D_3_ did not affect the expression of proteins of LAMP2A and HSC70 in ODM with or without leptin at 24 h; **f** relative quantitative analysis of proteins of LAMP2A and HSC70; **g** western blot analysis of JAK2/STAT3, PI3K/AKT, and MAPK/p38 in ODM at 1 h; **h** relative quantitative analysis of related signal pathway proteins. (*P < 0.05, **P < 0.01, ***P < 0.001 in comparison with the control group.)

To verify whether leptin inhibits CMA, we used qRT-PCR and western blot to detect the mRNA and protein levels of CMA-related genes LAMP2A and HSC70 in rBMMSCs. The results showed that the mRNA relative expression levels of LAMP2A and HSC70 decreased significantly in rBMMSCs, 8 h after different concentrations of leptin treatments (Fig. 3a), and the protein levels were similar to mRNA, 24 h after leptin treatments (Fig. 3b, c). However, 25(OH)D_3_ did not affect CMA in rBMMSCs with or without leptin (Fig. 3d, e, f).

### Leptin inhibited CMA through PI3K/AKT signal pathway

To explore the signal pathways involved in inhibiting CMA by leptin, we used western immunoblotting to quantify several signal pathway proteins related to leptin, including JAK2/STAT3, mitogen-activated protein kinase (MAPK)/p38, and PI3K/AKT signaling pathways. Our results showed that the expression level of p-AKT protein, but not p-STAT3, t-STAT3, p-P38, t-P38, and t-AKT, increased significantly, 1 h after rBMMSCs was stimulated with the increase in leptin concentration in ODM (Fig. 3g, h).

### The expression of megalin promoted by leptin through PI3K/AKT-dependent CMA was partially blocked by PI3K/AKT signal pathway inhibitor LY294002

**Fig. 4:**
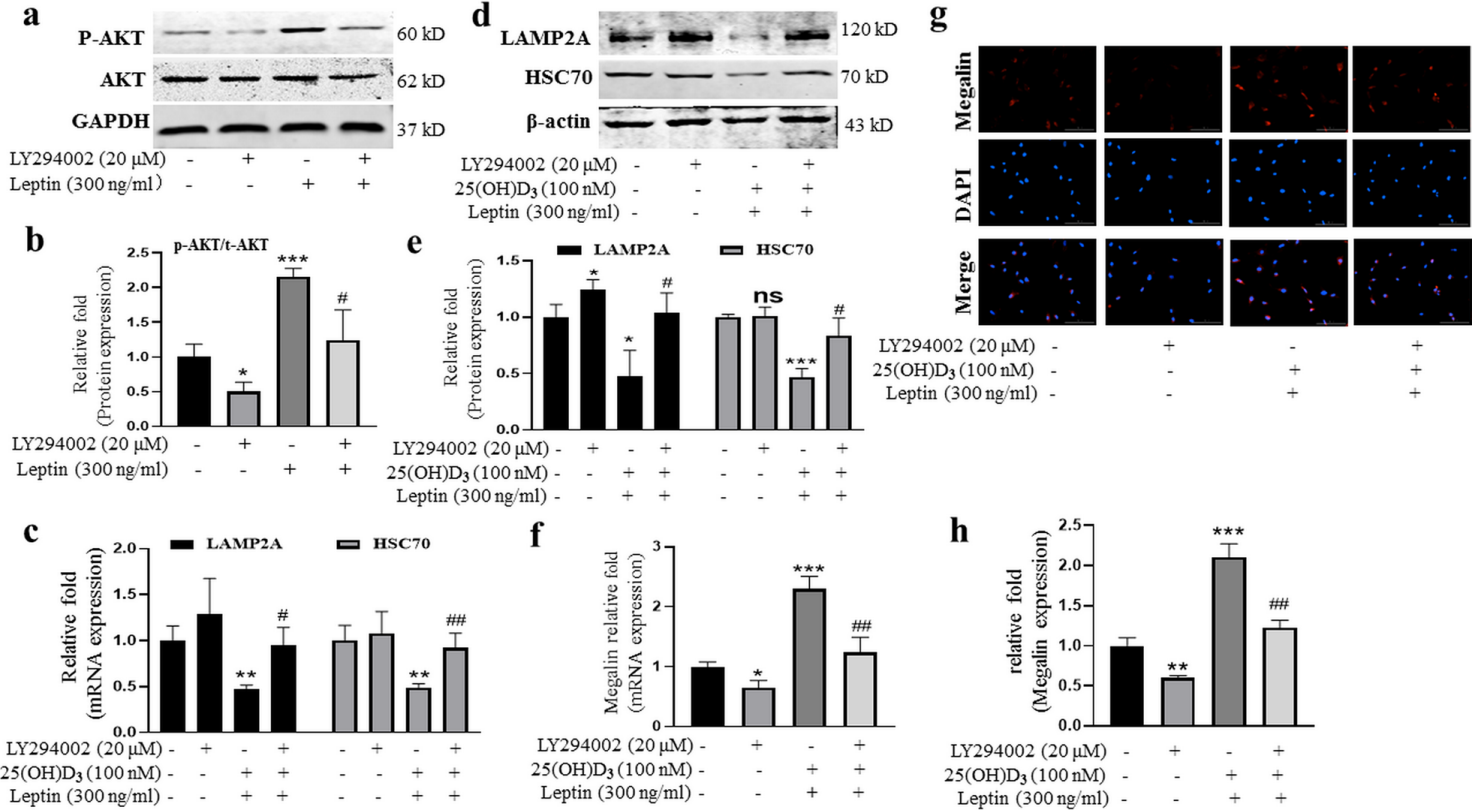
The expression of megalin promoted by leptin through PI3K/AKT-dependent CMA was partially blocked by PI3K/AKT signal pathway inhibitor LY294002 (20 μM). **a** Western blot analyses showed that the level of p-AKT was significantly decreased in leptin-treated rBMMSCs with the inhibitor in ODM at 1 h; **b** relative quantitative analysis of western blot analyses; **c** the mRNA relative expression of LAMP2A and HSC70 inhibited by leptin combined with 25(OH)D_3_ was partially rescued in ODM at 8 h; **d** the decreased protein levels of LAMP2A and HSC70 induced by leptin and 25(OH)D_3_, which were reversed by the inhibitor LY294002 (20 μM) at 24 h; **e** relative quantitative analysis of western blot analyses; **f** the mRNA relative expression of megalin by leptin with 25(OH)D_3_ + LY294002 was significantly decreased; **g** IF showed that the expression level of megalin enhanced by leptin and 25(OH)D_3_, which was significantly inhibited by the inhibitor LY294002 (20 μM) at 24 h; **h** relative quantitative analysis of megalin IF staining. (Scale bar = 100 μm, *P < 0.05, **P < 0.01, ***P < 0.001 in comparison with the control group; #*P* < 0.05, ##*P* < 0.01 in comparison with leptin + 25(OH)D_3_ group.)

To test whether PI3K/AKT signal pathway was involved in the expression of megalin, we used a PI3K/AKT signal pathway inhibitor, LY294002 (20 μM), to inhibit the expression of p-AKT and to measure the expression of CMA-related genes and megalin by qRT-PCR, IF, and western immunoblot. The PI3K/AKT signal pathway inhibitor LY294002 was added to the ODM 2 h earlier before leptin and 25(OH)D_3_. The results showed that the increased expression level of p-AKT caused by leptin was significantly inhibited by LY294002 (Fig. 4a, b), and mRNA relative expression of CMA-related gene (LAMP2A and HSC70) inhibited by leptin was partially rescued by inhibitor LY294002 at 8 h (Fig. 4c); the protein relative expression levels were similar to mRNA after 24-h treatments (Fig. 4d, e); the mRNA expression level of megalin enhanced by leptin was also significantly decreased at 8 h (Fig. 4f); IF analysis confirmed the up-regulation of megalin receptor caused by leptin was inhibited by the PI3K/AKT signal pathway inhibitor (Fig. 4g, h). These results showed that leptin promoted the expression of megalin via PI3K/AKT-dependent CMA.

### The ability of leptin synergistically with 25(OH)D_3_ promoted osteoblast differentiation of rBMMSCs was weakened by the PI3K/AKT signal pathway inhibitor

**Fig. 5:**
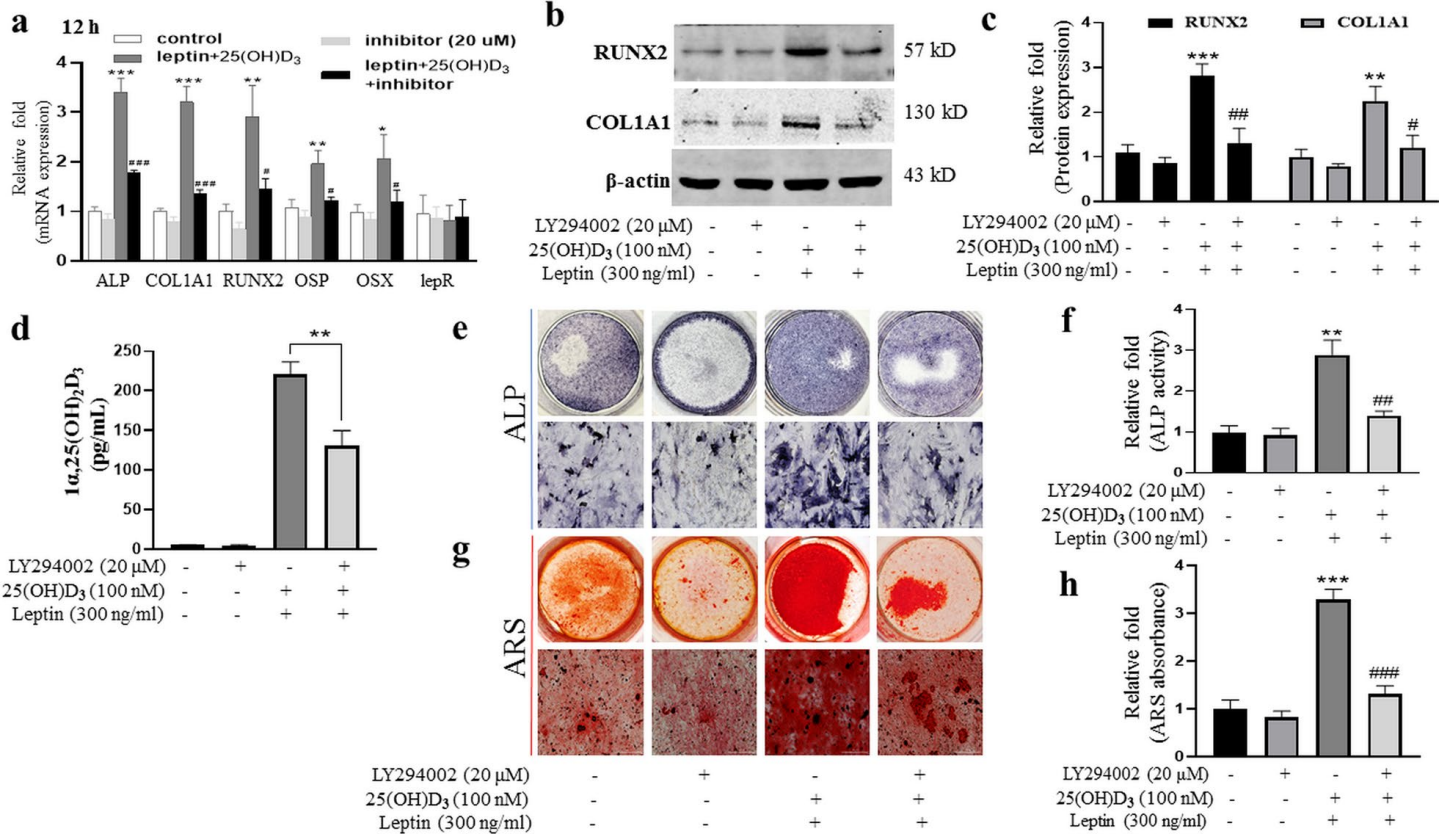
The ability of leptin synergistically with 25(OH)D_3_ promoted osteoblast differentiation of rBMMSCs was weakened by the PI3K/AKT signal pathway inhibitor. **a** The increased mRNA levels of ALP, COL1A1, RUNX2, OSX, and OSP induced by 25(OH)D_3_ combined with leptin were significantly decreased after the addition of LY294002 (20 μM) at 12 h; **b** the protein levels of RUNX2 and COL1A1 induced by leptin and 25(OH)D_3_ were significantly inhibited by the inhibitor LY294002 (20 μM) at day 3; **c** relative quantitative analysis of western blot analyses; **d** the synthesis of 1α,25(OH)_2_D_3_ by rBMMSCs with ODM at 24 h; **e** results of ALP staining; **f** the relative expression level of ALP activity; **g** results of ARS staining; **h** the relative expression level of ARS. (Scale bar = 200 μm, **P < 0.01, ***P < 0.001 in comparison with the control group; #*P* < 0.05, ##*P* < 0.01, ###*P* < 0.001 in comparison with leptin + 25(OH)D_3_ group.)

**Fig. 6:**
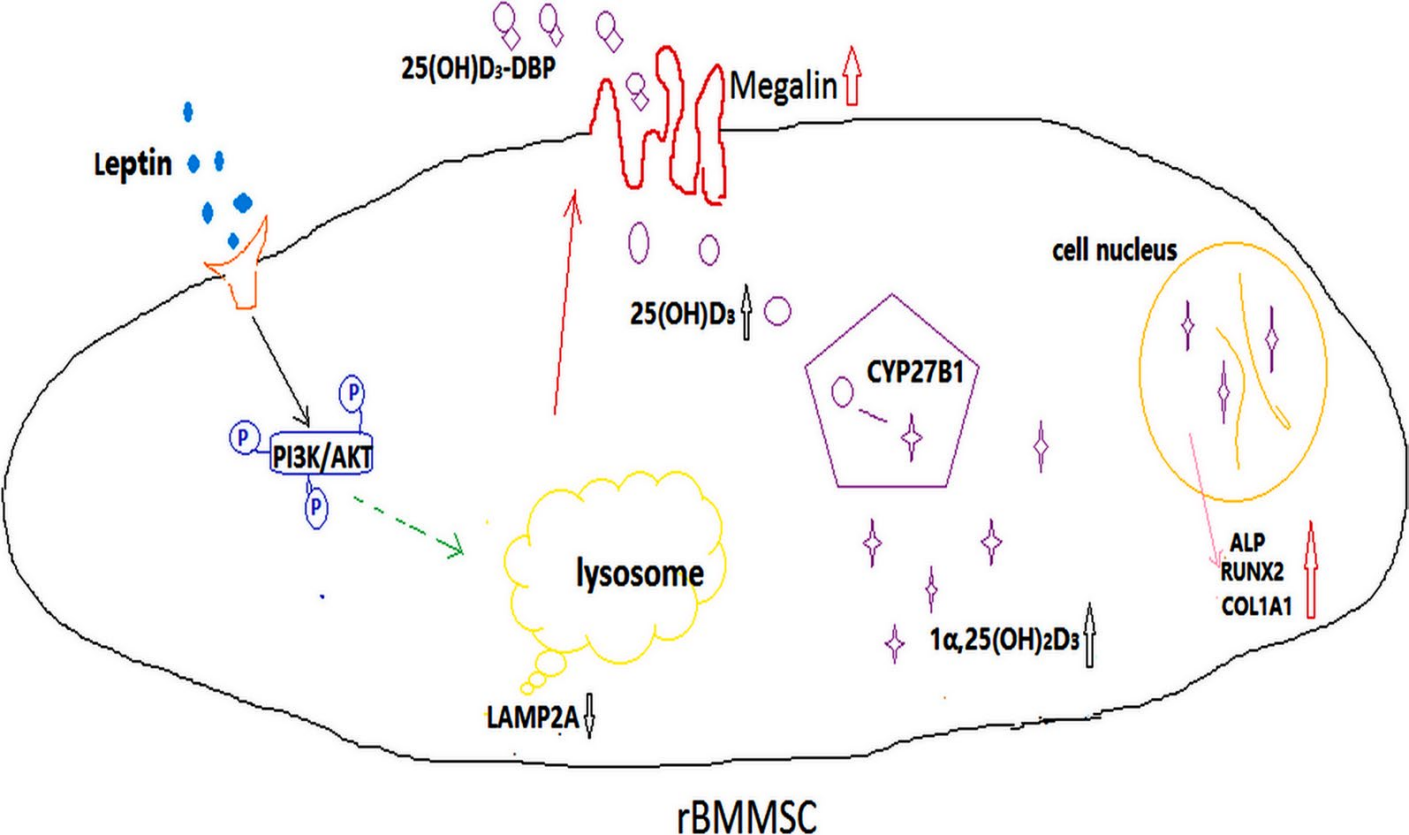
A summary diagram that leptin promotes megalin expression mediated by inhibition of CMA and synergistically with 25(OH)D_3_ to promote the osteoblast differentiation of rBMMSCs. As indicated by these studies, leptin inhibited the activity of CMA by activating PI3K/AKT signaling pathway to promote the expression of megalin, and then the quantity of 25(OH)D_3_ entering rBMMSCs was increased through increased megalin receptors. 25(OH)D_3_ was converted to 1α,25(OH)_2_D_3_ by 1α-hydroxylation of mitochondrial CYP27B1, and the synthesis of 1α,25(OH)_2_D_3_ was up-regulation and stimulated osteoblast differentiation of rBMMSCs

To test whether leptin synergistically with 25(OH)D_3_ on the stimulation of osteoblast differentiation via PI3K/AKT signal pathway, we used PI3K/AKT signal pathway inhibitor (LY294002, 20 μM). The increased mRNA expression levels of ALP, COL1A1, RUNX2, OSP, and OSX promoted by leptin synergistically with 25(OH)D_3_ were significantly inhibited by PI3K/AKT signal pathway inhibitor (LY294002, 20 μM) at 12 h, day 3 and 5 (Fig. 5a, Additional file 3e, f). Similarly, the protein expression levels of COL1A1 and RUNX2 were reduced at day 3 (Fig. 5b, c), and the synthesis of 1α,25(OH)_2_D_3_ was significantly lower in LY294002 with leptin + 25(OH)D_3_ group (Fig. 5d). Moreover, ALP activity, ALP staining, and ARS also showed that the ability of leptin synergistically with 25(OH)D_3_ promoted osteoblast differentiation of rBMMSCs was reduced after the addition of PI3K/AKT signal pathway inhibitor (LY294002, 20 μM) (Fig. 5e–h). Figure 6 is a summary diagram for the role of leptin in promoting megalin expression by inhibiting chaperone-mediated autophagy and enhancing osteoblast differentiation of rat bone marrow mesenchymal stem cells induced by 25(OH)D_3_.

## Discussion

1α,25(OH)_2_D_3_ plays an essential role in the osteoblast differentiation of BMMSCs, which may prevent age-related osteoporosis [21]. There are two main sources of vitamin D in the human body, one is intake and absorption from diet, and the other is skin exposure to ultraviolet rays. Vitamin D from both sources is hydroxylated to 25(OH)D_3_ by 25-hydroxylase (CYP2R1) in the liver and thereafter to active 1α,25(OH)_2_D_3_ by 1α-hydroxylase (CYP27B1) in the kidney [22]. Many studies found various extrarenal tissues such as skin, lymph nodes, colon, pancreas, adrenal medulla, dendritic cells, endothelial cells, brain, hypothalamus, placenta, bone cells, BMMSCs have 1α-hydroxylase (CYP27B1) [23–26]. Evidence shows that 25(OH)D_3_ binds to vitamin D binding protein (DBP) in blood and enters into the proximal tubular cell via the megalin receptor [27]. Megalin, a member of the low-density lipoprotein family, 600 kd, was expressed in renal proximal convoluted tubular epithelial cells. It reabsorbs glomerular-filtered proteins and acts as a nonspecific receptor for many proteins, including hormones, enzymes, drugs, and the 25(OH)D_3_-DBP complex [28]. Many extrarenal tissues also express megalin receptor, such as BMMSCs, alveoli, gallbladder, and placenta [29]. Our previous research and others showed that megalin is one of the key receptors for 25(OH)D_3_ in human BMMSCs and osteoblasts [16, 30], and the expression level of megalin determined the osteoblast differentiation potential of BMMSCs induced by 25(OH)D_3_. We found that the megalin expression levels in BMMSCs of some donors were relatively low, and 25(OH)D_3_ has little effects on the MSCs, which poor expression megalin [16]. In this study, we found that leptin regulates the megalin expression (Fig. 1), implying that the variation in megalin in different donors may be due to the different leptin levels among the donors, but further research is needed.

Studies have shown that 25(OH)D_3_ induces the osteoblast differentiation of BMMSCs at 250 nM, some even up to excess 500 nM, but not at 100 nM [31–34]; we hypothesized that it may relate to the significant variation of megalin in BMMSCs; the expression difference of megalin affects the uptake and utilization of 25(OH)_3_. At present, there is controversy about the normal physiological safe concentration of 25(OH)D_3_ in humans, which high concentration of 25(OH)D_3_ (> 125 nM) is thought to have side effects on the body [35–37]; therefore, this study focused on how to improve the sensitiveness of BMMSCs to 25(OH)D_3_. The concentration of 25(OH)D_3_ (100 nM) used in our study that did not significantly induce osteoblast differentiation of BMMSCs in vitro, but the co-stimulation of leptin and 25(OH)D_3_ significantly promoted the osteoblast differentiation of rBMMSCs showed with the results of qRT-PCR and western blots that the expression levels of osteoblastogenic genes ALP, COL1A1, RUNX2, OSX, and OSP are increased; ALP activity and ARS enhanced significantly after co-stimulation of leptin and 25(OH)D_3_.

We explored the mechanism of synergistic stimulation of leptin and 25(OH)D_3_ on the osteoblast differentiation of rBMMSCs. The results of qRT-PCR and IF showed that leptin enhanced the expression of megalin in rBMMSCs, but 25(OH)D_3_ had no effect on megalin; the ELISA results showed that leptin promoted 25(OH)D_3_ to enter into rBMMSCs. Our studies showed the importance of megalin expression in rBMMSCs for their osteoblast differentiation potential. Synergistic actions of 25(OH)D_3_ with leptin on osteoblast differentiation required up-regulation of megalin, the key receptor of the 25(OH)D3-DBP complex. When the megalin was up-regulated, more 25(OH)D_3_ was utilized to induce more osteoblast differentiation of rBMMSCs. These results are consistent to our previous report with human BMMSCs [16].

Leptin regulates energy metabolism and appetite through the hypothalamic leptin receptor [38]. Some evidence showed that leptin induces osteoblast differentiation of human BMMSCs [39], but others reported that leptin does not affect the osteoblast differentiation of rBMMSCs [40]. Studies have shown that bone mineral density in obese people is higher than that in ordinary people, especially in obese postmenopausal women, and the rate of bone loss slows down [41–43]. Leptin is positively correlated with obesity and positively affects bone mineral density, but the specific mechanism is still unclear [17]. Another study showed that the synthesis and secretion of leptin was increased in obese people [44]. Leptin receptor is highly expressed in BMMSCs, which include precursor cells of osteoblasts [45]. In our study, we found that leptin or 25(OH)D_3_ had no significant effect on the expression of leptin receptor. PET imaging showed that 15% labeled leptin concentrated in red bone marrow in rhesus monkeys and binding at high level in bone marrow tissue of mice via leptin receptor [46].

Most importantly, we discovered that leptin inhibited CMA through the PI3K/AKT signal pathway to promote the expression of megalin, and the expression level of genes (LAMP2A and HSC70) was suppressed. The results of qRT-PCR and western blots showed that PI3K/AKT inhibitor could reverse the inhibited effect of CMA caused by leptin, and the expression of megalin was down-regulated. ELISA results showed that the entry of 25(OH)D_3_ into rBMMSCs was decreased, and the synergistic effect of leptin and 25(OH)D_3_ on osteoblast differentiation of rBMMSCs was inhibited. There are three types of autophagy, including macroautophagy, microautophagy, and CMA; similar to the results of our study, leptin is an inhibitor of autophagy to promote apoptosis of chondrocytes through PI3K/AKT signal pathway during osteoarthritis pathogenesis [47]. In a chronic overpressure mouse model, leptin repressed the cardiac autophagy by the PI3K/AKT signal pathway and led to cardiac dysfunction [48]. These results indicated that leptin is an inhibitor of PI3K/AKT-dependent CMA; leptin promoted the expression of megalin by inhibiting CMA.

CMA maintains the physiological function of cells, which can degrade specific cellular proteins in time through the lysosome. These proteins contain specific amino acid sequences KFERQ; about 40% of the proteins in the mammalian contain typical KFERQ sequences. Heat-shock protein HSC70 recognizes and binds the specific amino acid sequence of these proteins, unfolds the substrate protein, then enters the lysosome through the LAMP2A receptor on the lysosomal membrane for subsequent degradation [49]. LAMP2A receptor is the key rate-limiting part of CMA and the specific marker of CMA. The number and activity of LAMP2A determine the level of CMA. CMA is involved in regulating glucose and lipid metabolism, DNA repair, cell reprogramming, and cell response to stress [19]. In a study by Li et al. [50], inhibition of the PI3K/AKt/mTOR signaling pathway was protective in acute liver failure by promoting CMA. Our results showed that leptin significantly reduced the expression of LAMP2A and that the PI3K/AKT signal pathway inhibitor (LY294002) partially rescued the inhibited CMA indicated that leptin induced megalin via inhibiting CMA through PI3K/AKT signal pathway. Our studies do not identify protein substrates involved in CMA, but they support continued investigations to study the protein substrate involved in CMA that affects the expression of megalin.

## Conclusion

In sum, these data suggest the mechanism by which there is synergy between leptin and 25(OH)D_3_ to promote osteoblast differentiation. Leptin promoted the expression of megalin by inhibiting CMA, increased the utilization of 25(OH)D_3_ by rBMMSCs, and enhanced the ability of 25(OH)D_3_ to induce osteoblast differentiation of rBMMSCs. PI3K/AKT is at least partially involved in the regulation of CMA. These data indicate the importance of megalin in BMMSCs for vitamin D’s role in skeletal health.

## Supplementary Information

**Additional file 1**. Identification of rBMMSCs and morphological and microstructure changes during osteoblast differentiation. **a** Morphology of BMMSCs at P3 under the inverted microscopy (scale bar = 1000 μm). **b** Flow cytometry analysis of surface markers (CD29, CD90, CD44, CD45) of BMMSCs. **c** The morphological changes of rBMMSCs during osteoblast differentiation at day 10 under the inverted microscopy (scale bar = 200 μm). **d** The microstructure changes of rBMMSCs during osteoblast differentiation at day 10 under the transmission electron microscope (scale bar = 2 μm)

**Additional file 2**. The cytotoxicity effects of leptin and 25(OH)D_3_ on rBMMSCs proliferation. **a** Leptin with different concentrations on the cytotoxicity effects of rBMMSCs proliferation at day 3. **b** 25(OH)D_3_ with different concentrations on the cytotoxicity effects of rBMMSCs proliferation at day 3. (*P < 0.05, **P < 0.01, ***P < 0.001 in comparison with the control group.)

**Additional file 3**. Leptin synergistically with 25(OH)D_3_ to induce osteoblast differentiation of rBMMSCs and was inhibited by inhibitor LY294002. **a**, **b** Relative mRNA expression of osteoblastogenic genes (ALP, COL1A1, RUNX2, OSX, and OSP) at days 3 and 5. **c**, **d** The expression of protein of leptin receptor at day 3. **e**, **f** The effect of inhibitor on the mRNA expression of osteoblastogenic genes at day 3 and 5. (*P < 0.05, **P < 0.01, ***P < 0.001 in comparison with the control group. #*P* < 0.05, ##*P* < 0.01 in comparison with leptin + 25(OH)D_3_ group.)

## Data Availability

The data that support the findings of this study are available from the corresponding author upon reasonable request.

## Abbreviations

BMMSCs: Bone marrow mesenchymal stem cells
1α,25(OH)_2_D_3_: 1α,25-Dihydroxy-cholecalciferol
25(OH)D_3_: 25-Hydroxycholecalciferol
ARS: Alizarin Red Staining
ALP: Alkaline phosphatase
ODM: Osteogenic differentiation medium
qRT-PCR: Quantitative real-time polymerase chain reaction
GAPDH: Glyceraldehyde-3-phosphate dehydrogenase
RUNX2: Runt-related transcription factor 2
COL1A1: Collagen type I
OSX: Osterix
OSP: Osteopontin
LAMP2A: Lysosomal-associated membrane protein 2A
HSC70: Heat-shock cognate 71 kDa protein
lepR: Leptin receptor
