# Supplementary figures and images for: Synergistic stimulation of osteoblast differentiation of rat mesenchymal stem cells by leptin and 25(OH)D3 is mediated by inhibition of chaperone-mediated autophagy
Source: Stem Cell Res Ther. 2021 Oct 30;12:557. doi: 10.1186/s13287-021-02623-z (PMC8557551; doi:10.1186/s13287-021-02623-z)

**a**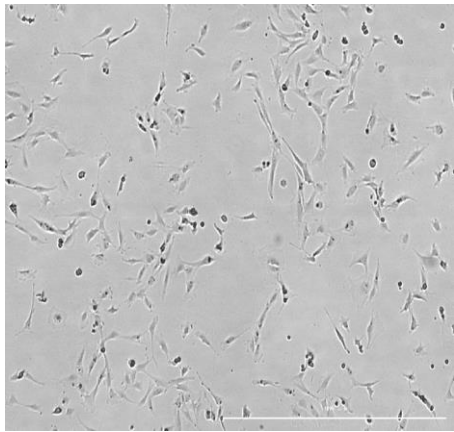**b**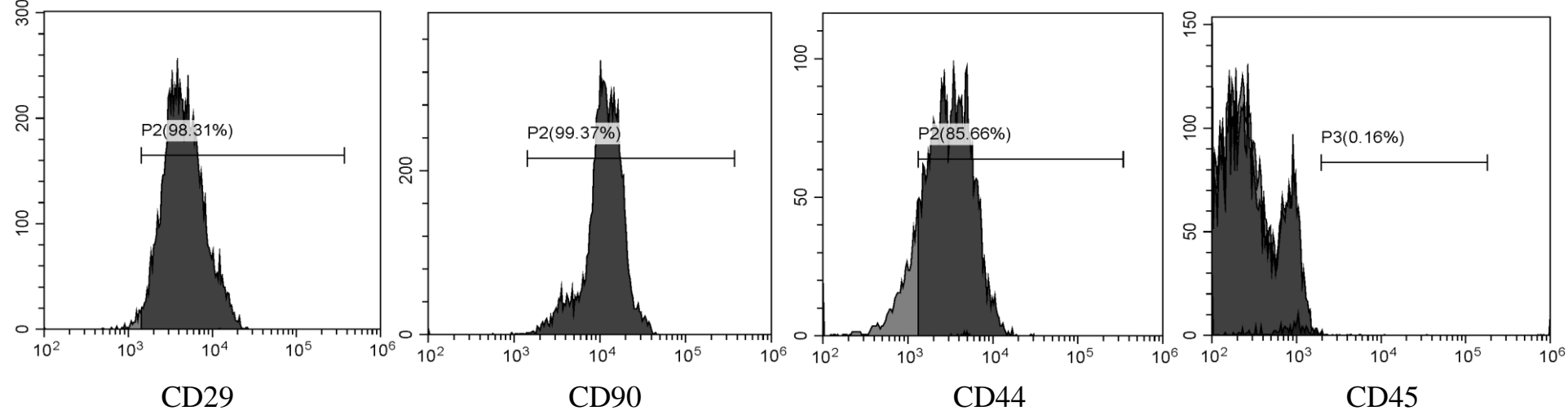**c**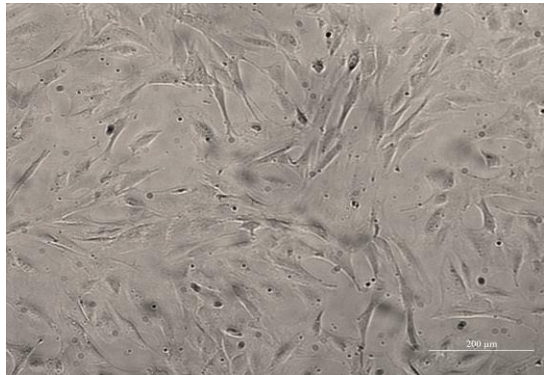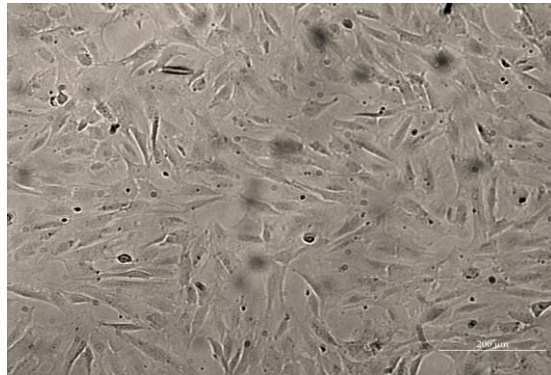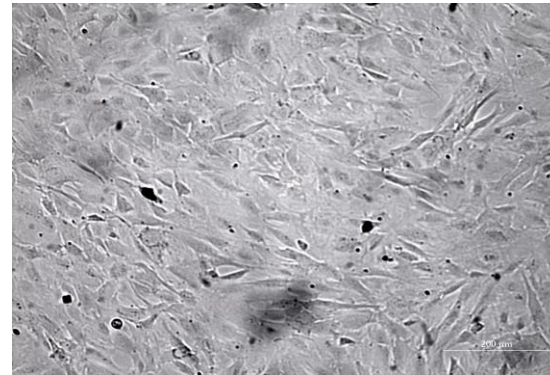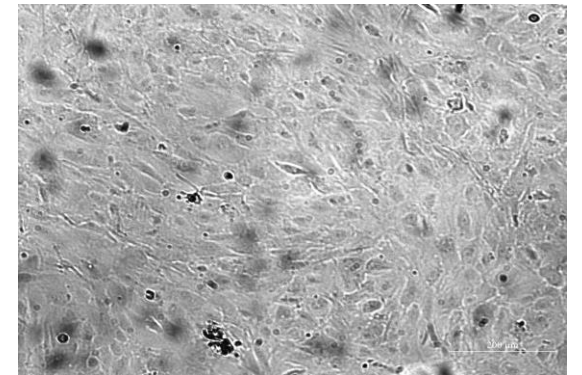**d**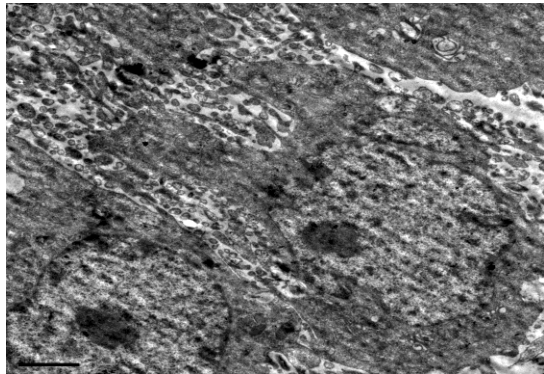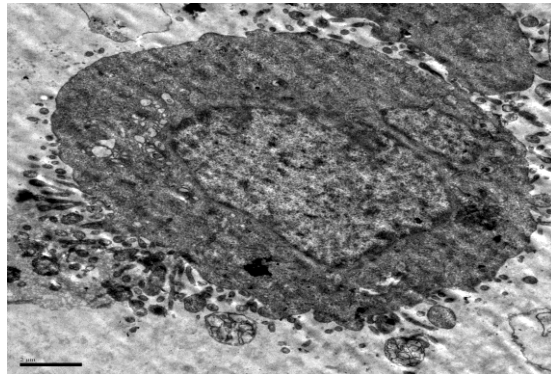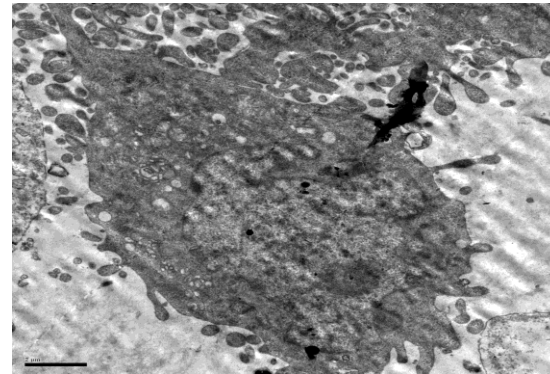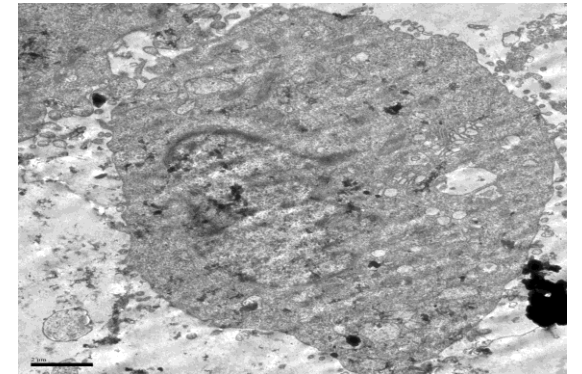**control****leptin****25(OH)D<sub>3</sub>****leptin + 25(OH)D<sub>3</sub>**

Supplement: Supplementary file 1 — Additional file 1. Identification of rBMMSCs and morphological and microstructure changes during osteoblast differentiation. a Morphology of BMMSCs at P3 under the inverted microscopy (scale bar = 1000 μm). b Flow cytometry analysis of surface markers (CD29, CD90, CD44, CD45) of BMMSCs. c The morphological changes of rBMMSCs during osteoblast differentiation at day 10 under the inverted microscopy (scale bar = 200 μm). d The microstructure changes of rBMMSCs during osteoblast differentiation at day 10 under the transmission electron microscope (scale bar = 2 μm) [file 13287_2021_2623_MOESM1_ESM.pdf]

**a****Leptin (3 d)**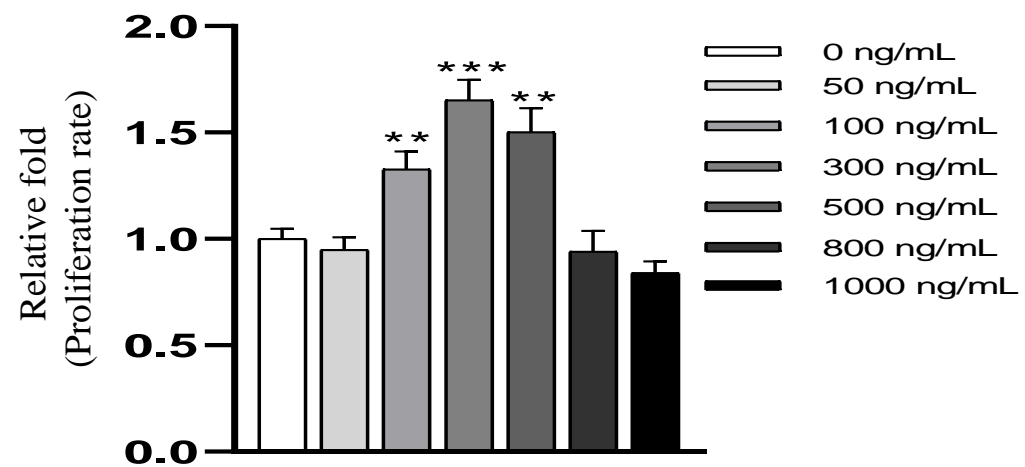**b****25(OH)D<sub>3</sub> (3 d)**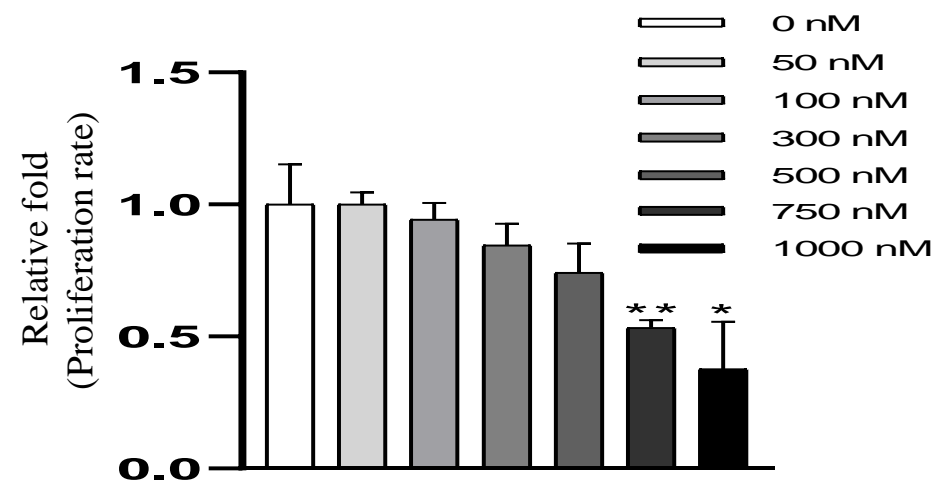

Supplement: Supplementary file 2 — Additional file 2. The cytotoxicity effects of leptin and 25(OH)D3 on rBMMSCs proliferation. a Leptin with different concentrations on the cytotoxicity effects of rBMMSCs proliferation at day 3. b 25(OH)D3 with different concentrations on the cytotoxicity effects of rBMMSCs proliferation at day 3. (*P < 0.05, **P < 0.01, ***P < 0.001 in comparison with the control group.) [file 13287_2021_2623_MOESM2_ESM.pdf]

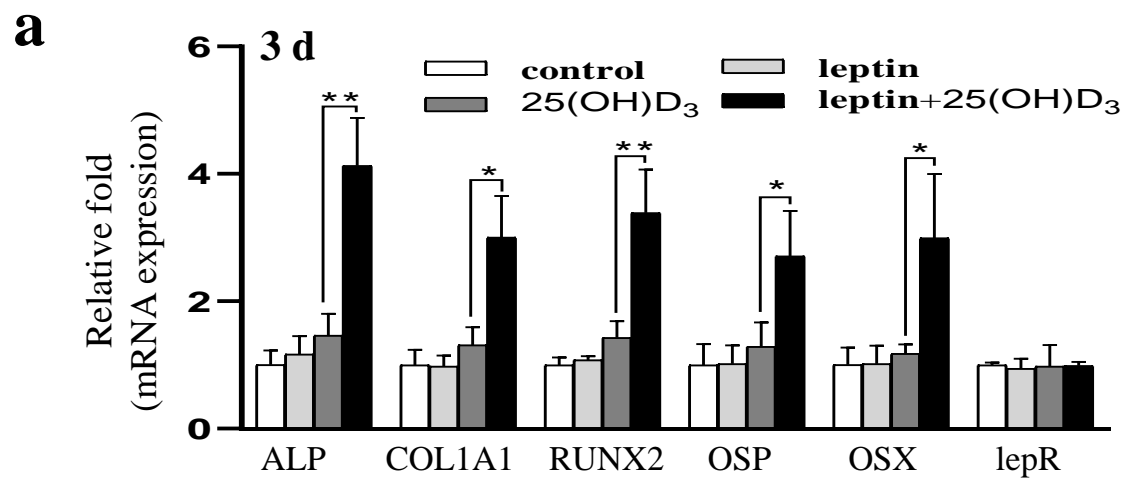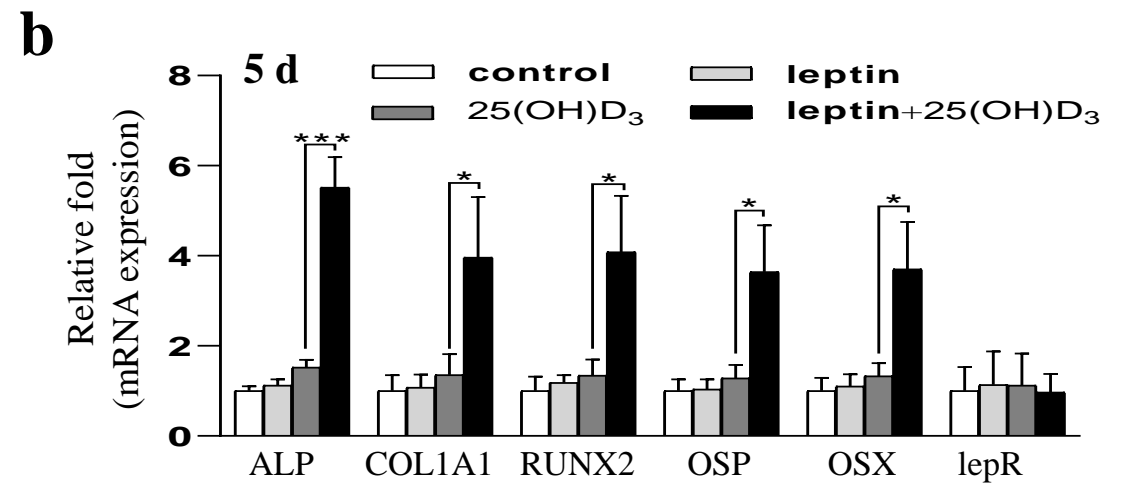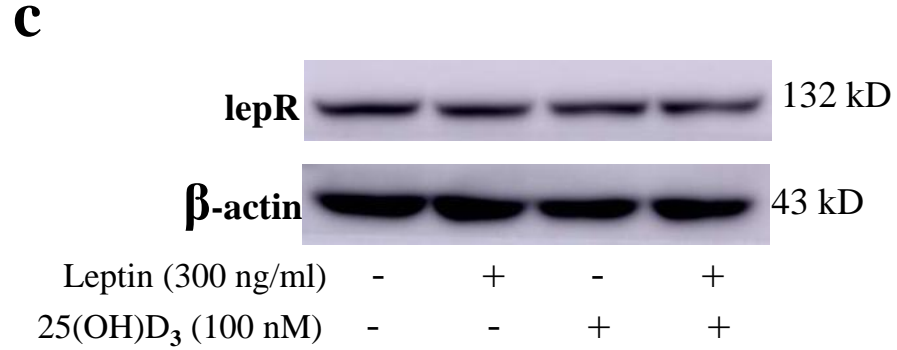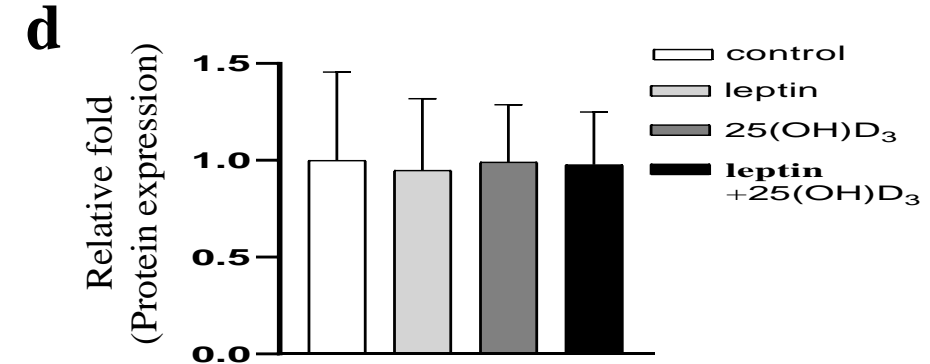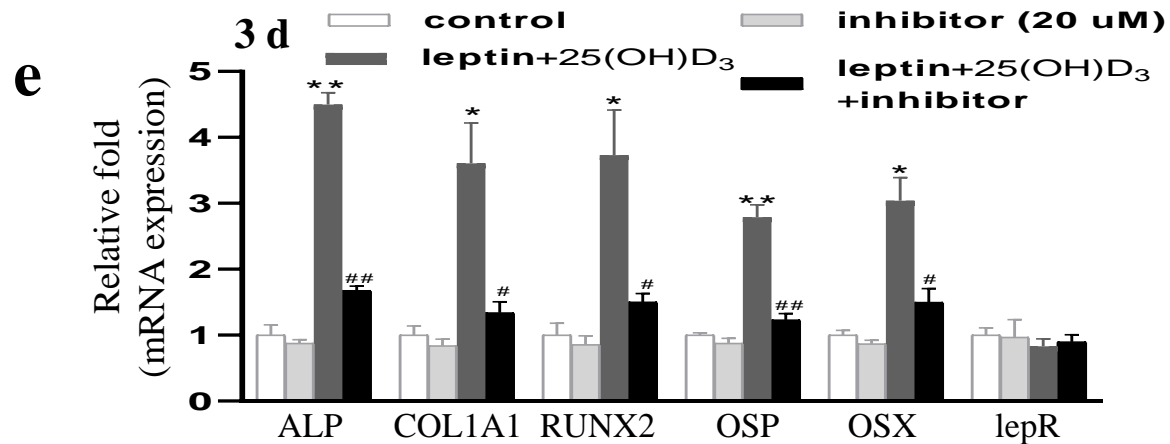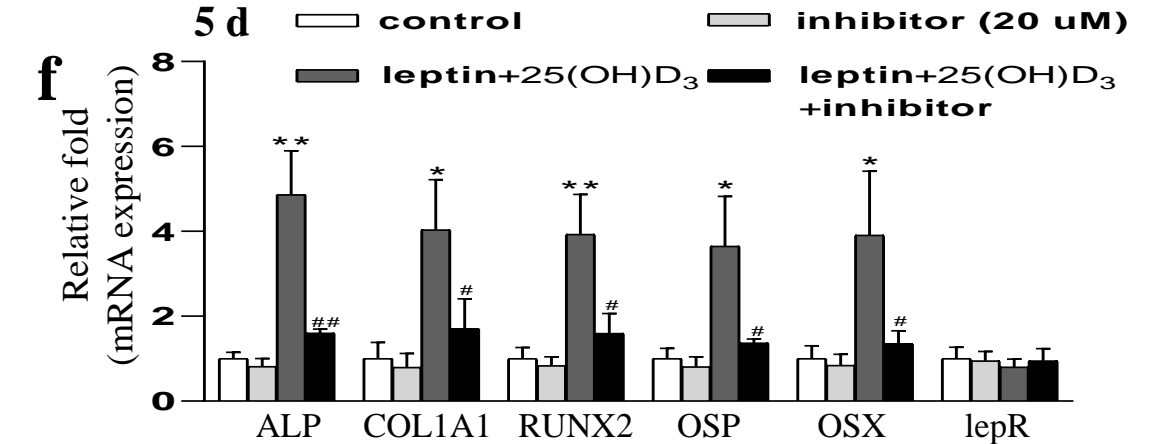

Supplement: Supplementary file 3 — Additional file 3. Leptin synergistically with 25(OH)D3 to induce osteoblast differentiation of rBMMSCs and was inhibited by inhibitor LY294002. a, b Relative mRNA expression of osteoblastogenic genes (ALP, COL1A1, RUNX2, OSX, and OSP) at days 3 and 5. c, d The expression of protein of leptin receptor at day 3. e, f The effect of inhibitor on the mRNA expression of osteoblastogenic genes at day 3 and 5. (*P < 0.05, **P < 0.01, ***P < 0.001 in comparison with the control group. #P < 0.05, ##P < 0.01 in comparison with leptin + 25(OH)D3 group.) [file 13287_2021_2623_MOESM3_ESM.pdf]
